# Systematic Analysis of Gene Expression in Lung Adenocarcinoma and Squamous Cell Carcinoma with a Case Study of *FAM83A* and *FAM83B*

**DOI:** 10.3390/cancers11060886

**Published:** 2019-06-25

**Authors:** Ling Cai, Danni Luo, Bo Yao, Donghan M. Yang, ShinYi Lin, Luc Girard, Ralph J. DeBerardinis, John D. Minna, Yang Xie, Guanghua Xiao

**Affiliations:** 1 Quantitative Biomedical Research Center, Department of Population and Data Sciences, Simmons Comprehensive Cancer Center, UT Southwestern Medical Center, 5323 Harry Hines Blvd, Dallas, TX 75390, USA; ling.cai@utsouthwestern.edu (L.C.); Danni.Luo@UTSouthwestern.edu (D.L.); Bo.Yao@UTSouthwestern.edu (B.Y.); Donghan.Yang@UTSouthwestern.edu (D.M.Y.); Shinyi.Lin@UTSouthwestern.edu (S.L.); yang.xie@utsouthwestern.edu (Y.X.); 2 Howard Hughes Medical Institute and Children’s Medical Center Research Institute, UT Southwestern Medical Center, 5323 Harry Hines Blvd, Dallas, TX 75390, USA; Ralph.Deberardinis@UTSouthwestern.edu; 3 Hamon Center for Therapeutic Oncology Research, Department of Pharmacology, Department of Internal Medicine, Simmons Comprehensive Cancer Center, University of Texas Southwestern Medical Center, 5323 Harry Hines Blvd, Dallas, TX 75390, USA; luc.girard@utsouthwestern.edu (L.G.); John.Minna@UTSouthwestern.edu (J.D.M.)

**Keywords:** lung cancer, gene expression difference between tumor and normal, survival association analysis, systematic analysis, meta-analysis, FAM83

## Abstract

Introduction: In our previous study, we constructed a Lung Cancer Explorer (LCE) database housing lung cancer-specific expression data and clinical data from over 6700 patients in 56 studies. Methods: Using this dataset of the largest collection of lung cancer gene expression along with our meta-analysis method, we systematically interrogated the association between gene expression and overall survival as well as the expression difference between tumor and normal (adjacent non-malignant tissue) samples in lung adenocarcinoma (ADC) and lung squamous cell carcinoma (SQCC). A case study for *FAM83A* and *FAM83B* was performed as a demonstration for hypothesis testing with our database. Results: We showed that the reproducibility of results across studies varied by histological subtype and analysis type. Genes and pathways unique or common to the two histological subtypes were identified and the results were integrated into LCE to facilitate user exploration. In our case study, we verified the findings from a previous study on *FAM83A* and *FAM83B* in non-small cell lung cancer. Conclusions: This study used gene expression data from a large cohort of patients to explore the molecular differences between lung ADC and SQCC.

## 1. Introduction

Lung cancer is the leading cause of cancer-related death worldwide. Over the past few decades, genome-wide cancer transcriptomic and mutational studies have produced a massive amount of data, allowing researchers to better understand lung cancer development at the molecular level, and has enabled the discovery of biomarkers that facilitate cancer treatments for individual patients (“precision medicine”). Such data have been deposited into public data repositories, re-annotated, and collected into many cancer databases.

Lung Cancer Explorer (LCE) is a data commons tool for the research community: We recently described our development of a new data commons, Lung Cancer Explorer (LCE), with a web application (http://lce.biohpc.swmed.edu/), populated by a centralized lung cancer database [1]. Our database houses the largest collection of lung tumor expression data from 23 different platforms (including both microarray and RNA-Seq) collected from 56 studies for over 6700 patients and enriched with rigorously curated clinical data (please see the LCE article’s Methods Section for further details on our curation of clinical data) [1].

This user-friendly open web portal provides several easy, but versatile analysis tools. These tools include meta-analysis, which enables users to gain a quick overview of the results from all datasets while combining the statistical power from multiple datasets as well as individual dataset-based analyses that allow for more flexibility and customization from the user. However, of greatest importance, LCE is configured so that users may easily download the processed and standardized clinical and gene expression data to perform their own analyses of interest.

The functionality of LCE is designed for the analyses of individual genes, which makes the tools useful for hypothesis testing when users identify putative gene(s) of interest. Nevertheless, we also understand the importance of systematic analysis at the genome-wide level. Such results can be used to drive hypothesis generation. Users can also observe how their gene of interest compares to all of the other genes in the same analysis and how robust the analysis is in identifying significant hits.

Lung adenocarcinoma (ADC) and lung squamous cell carcinoma (SQCC) are two dominant histological subtypes that together account for 70% of all lung cancer cases [2]. Although under the same umbrella of non-small cell lung cancer (NSCLC), ADC and SQCC differ in disease etiology. While SQCC mostly develops in smokers, ADC is common in people who have never smoked [3]. ADC and SQCC also exhibit different mutation spectra and hence demand different therapeutic strategies [4]. Recognizing these differences, we analyzed ADC and SQCC separately, then compared and contrasted the results to gain more insights into their commonalities and differences.

In this paper, we present the results from the systematic analyses of ADC and SQCC that examined gene expression association with patient survival outcomes as well as tumor vs. normal (adjacent non-malignant tissue) gene expression difference. We highlighted some interesting observations from the pathway analyses. These results have also been uploaded to Lung Cancer Explorer under “SYSTEMATIC ANALYSIS RESULTS”, which allows users to explore and answer their own research questions. We also present a case study of *FAM83A* and *FAM83B* to validate the findings from a recent investigation that proposed to use them as prognostic biomarkers and potential new therapeutic targets in NSCLC [5]. Through the meta-analyses of gene-expression difference in tumor vs. normal, in ADC vs. SQCC, and in mutants vs. WT for *EGFR* or *KRAS* as well as the meta-analyses of survival association and gene–gene correlation, we present multiple lines of evidence generated from our lung cancer database to strengthen the existing findings.

## 2. Materials and Methods

### 2.1. Lung Cancer Database

Construction of the lung cancer database was described in detail in our previous publication [1]. The processed data are available for download from http://lce.biohpc.swmed.edu/.

### 2.2. Meta-Analysis

For tumor vs. normal differential expression meta-analysis, the R package metafor [6] was used to calculate the summary standardized mean difference (tumor vs. normal) using Hedges’ G as an effective size metric. Studies included for meta-analysis had to have at least 10 samples in each group, and meta-analysis was only performed for genes with data available from at least three qualifying studies. The R package meta [7] was used to calculate the summary HR from the HRs of individual datasets in the survival association meta-analysis, and summary Pearson correlation for the correlation meta-analysis. For survival association meta-analysis, only studies with at least 10 samples that had survival data were included, and meta-analysis was only performed for genes with data available from at least three qualifying studies. The Cox Proportional Hazard model was applied for expression data and further standardized to have a mean of 0 and standard deviation of 1 across all cancer samples of a study.

### 2.3. Pathway Enrichment Analysis

For pathway enrichment analysis, the R package fgsea [8] was used for gene set libraries downloaded from MSigDB [9].

### 2.4. Ethics Approval and Consent to Participate

The University of Texas Southwestern Institutional Review Board granted approval for this research (IRB#: STU 072016-028). This research only involved data being downloaded and curated from the public domain. All methods were performed in accordance with the relevant guidelines and regulations.

### 2.5. Availability of Data and Material

All of the processed data are available from http://lce.biohpc.swmed.edu/.

## 3. Results

### 3.1. Quality Control and Reproducibility across Different Studies

It is difficult to predict the likelihood of replicating the findings from one study in another cohort or study. Many factors contribute to the rise of the non-reproducibility of transcriptomic conclusions between different studies and include differences in patient demographics, clinical data curation, clinical care, inclusion/exclusion criteria, sample procurement procedures, and data acquisition platforms, among many others. In fact, we observed substantial study-to-study heterogeneity in the data we collected into our lung cancer database. Here, we explore this issue as exemplified by the results from four types of analyses.

#### 3.1.1. Comparison between Pooled-Sample Analysis and Meta-Analysis

Simply pooling samples from related datasets together seems to be a convenient way to boost the statistical power of the analysis. As a result, tools employing such an approach have been popular for cancer studies [10]. However, this can be a poor choice when it involves pooling samples from individual studies with different characteristics, since this kind of pooling has the potential to inflate the statistical significance, leading to false positive hits [11]. Meta-analysis, on the other hand, weighs studies differently and alleviates such problems.

As a concrete demonstration of how such pooling can influence the results of lung cancer transcriptomic studies, we directly compared the pooled sample analysis and meta-analysis approaches. To do this, we randomly permutated the “tumor” and “normal” tissue sample labels in the tumor vs. normal expression difference analysis for ADC and examined the results. After the random permutation, there should not be true positive hits as the sample labels are randomly assigned as either tumor or normal. As a result, the p-values should be uniformly distributed (under the Null hypothesis), which indeed was the case for the results from the meta-analysis, as seen in the Quantile–Quantile (QQ) plot (Figure S1A). The pooled-sample analysis, however, drastically inflated the significance of the results from this permutation test, leading to over one-third of the results for 25,734 genes remaining statistically significant. We noted that this significance remained even after a conservative multiple comparison adjustment by the Bonferroni correction. These results suggest that a pooled-sample analysis gives rise to many false positive hits when compared to a meta-analysis. In contrast, when we applied the meta-analysis approach to our actual data (samples remained correctly labeled as “tumor” and “normal”), we now found a large number of significant results (Figure S1B).

Compared with an analysis of individual datasets, a meta-analysis addresses the reproducibility issue from different studies. It provides not only the effect size of interest summarized from multiple studies, but also the study heterogeneity measure that reflects the cross-study generalizability.

#### 3.1.2. Quality Control for Clinical Variables and Survival Outcomes

**Figure 1:**
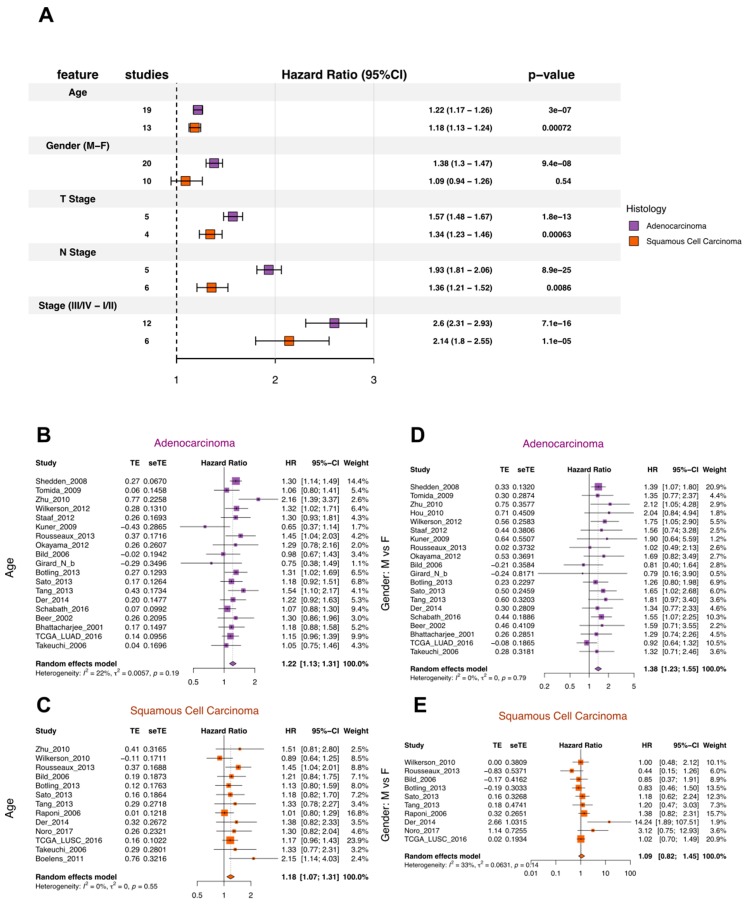
Meta-analysis of survival association for demographics and tumor stages. (**A**) Summary statistics from the meta-analysis of univariate survival association by the Cox-PH model for demographic and tumor stage variables in Adenocarcinoma (ADC) and squamous cell carcinoma (SQCC). (**B**,**C**) Forest plots showing the meta-analysis results of the survival association for age (10 years is used as 1 unit for age). Increased age was associated with worse survival in both ADC (**B**) and SQCC (**C**). (**D**,**E**) Forest plots showing the meta-analysis results of the survival association for gender. Male gender was significantly associated with worse survival in ADC (**D**) but not SQCC (**E**).

In establishing our lung cancer database, we placed great emphasis on the standardization of gene expression data and clinical data from different studies. These “harmonization” efforts empowered us to take the meta-analysis approach to conduct our systematic analysis. As a “quality control” check on our data and the meta-analysis approach, we first conducted a set of survival association analyses purely based on the clinical data in our data collection. For ADC and SQCC, we selected five clinical variables: age, gender, T-stage, N-stage, and TNM stage, which we knew had to have relevant survival differences, and performed a survival association meta-analysis using a univariate Cox proportional-hazards (Cox-PH) regression model (Figure 1). As expected, we saw worse survival with increases in age, T-stage, N-stage, and TNM-stage. The gender-associated difference was only significant in ADC, where males had worse prognosis (Figure 1). Interestingly, this observation matched a previous report based on 4618 patients diagnosed between 1997 and 2002, where worse prognosis for males was also only seen for ADC, but not SQCC after controlling for stage, pack years, and treatment in a multivariate model [12]. These results showed that the clinical data identified the expected prognostic clinical factors for overall survival analysis.

#### 3.1.3. Principal Component Analysis of the Transcriptomic Data Comparing Tumor and Normal Lung

Principal component analysis is commonly used to visualize the separation of tumor and normal samples in cancer transcriptomic studies, however, which genes are expressed at different levels in lung cancer when compared to normal lung? We also performed this analysis with our dataset collection. For twenty-two studies, the tumor and normal tissue samples were well separated on the first and second principal components. However, we did find exceptions in two studies, indicating that additional factors other than tissue type exerted a stronger influence in driving the transcriptomic differences across all samples (Figure S2).

#### 3.1.4. Reproducibility in Tumor vs. Normal Expression Difference and Gene Expression-Survival Association

In this paper, we focused on two analyses: gene expression differences between tumors and normal lung tissue, and associations between gene expression and survival. To assess the reproducibility across studies for each dataset, we calculated the standardized mean difference between the tumor samples and normal tissue samples as well as the hazard ratio from a Cox-PH model that measured the association between gene expression and patient survival. Next, we computed the pairwise correlation among studies on all genes (Figure S3). Highly positive correlations were observed among the tumor vs. normal standardized mean difference for both ADC (median of correlation coefficient at 0.75) and SQCC (median at 0.63), suggesting that the results are highly reproducible across studies, regardless of the histological subtype (Figure S3A,B). However, results from the survival association analysis across studies are much less reproducible; only modestly positive correlations (median at 0.177 for 231 pairwise correlations from 22 studies) were observed for the ADC datasets, (Figure S3C) whereas the reproducibility was generally poor for the SQCC studies (median at 0.025 for 136 pairwise correlations from 17 studies), except between two studies originating from the same research group for patients from MD Anderson within a similar time frame (Sato et al., 2013 [13] and Tang et al., 2013 [14]) (Figure S3D). Notably, some studies such as Hou et al., 2010 [15] have had good reproducibility in tumor vs. normal expression difference analysis, but the reproducibility was still poor in the survival association analysis.

### 3.2. Tumor vs. Normal Expression Difference in ADC and SQCC

**Figure 2:**
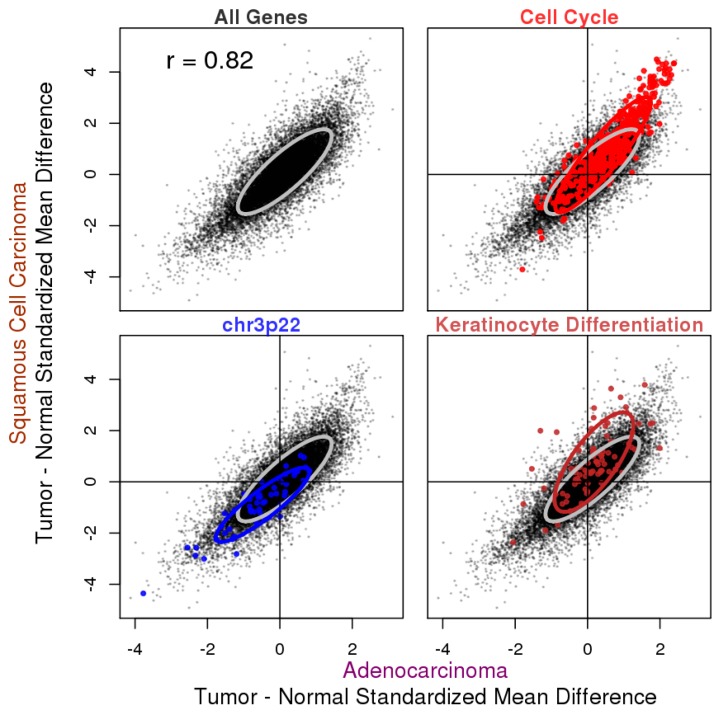
Tumor: the normal standardized mean expression difference in ADC and SQCC with selected genes highlighted. Summary tumor: the normal standardized mean difference from the meta-analysis was plotted for all genes, with results from ADC on the x-axis and results from SQCC on the y-axis. The Pearson correlation coefficient for all genes was 0.82. Genes from three selected gene sets were highlighted: the cell cycle gene set from REACTOME_CELL_CYCLE in c2.cp curated the canonical pathway gene sets collection, chr3p22 genes were from the c1 positional gene sets collection and keratinocyte differentiation genes were from GO_KERATINOCYTE_DIFFERENTIATION in the c5.bp GO biological process gene sets collection in MSigDB. Note that the majority of cell cycle genes were upregulated and the majority of chr3p22 genes were downregulated in the tumors of both histological subtypes, whereas the keratinocyte differentiation genes were specifically upregulated in the SQCC tumors. Ellipsoidal boundary wrapped around the 75% highest density/minimum volume for all genes (gray) or selected genes (non-gray colors).

In the tumor vs. normal expression difference analysis, we produced the summary standardized difference (SMD) between the tumor and normal tissues from the meta-analysis of ADC and SQCC datasets (Tables S1 and S2). When comparing the SMDs from the two histological subtypes, we found that they were highly consistent with a Pearson correlation coefficient of 0.82 (Figure 2). We performed pathway analysis by Fast Gene Set Enrichment Analysis (fgsea) [8] using selected gene set libraries from MSigDB [9] to seek biological interpretations of genes that are concertedly up- or downregulated together (Tables S3 and S4). Selected interesting hits from the results are highlighted in Figure 2 (see also Figure S4 for statistical summary).

Cell cycle genes are predominantly upregulated in tumors when compared to normal tissues, agreeing with the idea that chronic and often uncontrolled proliferation is a hallmark of cancer [16]. Genes on cytogenetic band chr3p22, on the other hand, are predominantly downregulated in cancer samples, in line with the previous report that allele loss on chromosome arm 3p is one of the most frequent and earliest known genetic events in lung cancer pathogenesis [17]. We further examined the status of tumor–normal gene expression difference for all genes on chromosome 3p and found an overall decrease (Figure S5A). In addition, while our analysis picked up chr3p22, studies have mapped the tumor suppressor genes (TSGs) onto 3p21.3. Since the cytogenetic band gene set library from MSigDB has a rather coarse resolution, we selected eight candidate TSGs designated 3p21.3 by a previous report [18] and examined their expression difference in tumors and normal samples in ADC and SQCC (Figure S5B,C). We found that all eight genes were downregulated in SQCC and five genes were downregulated in ADC.

Aside from the consistent changes for both ADC and SQCC, we also observed histology-specific changes: genes associated with keratinocyte differentiation were only enriched in the tumor over-expressed genes for SQCC, but not ADC, as keratinization is a prominent feature of SQCC but not ADC.

### 3.3. Gene Expression Association with Overall Survival in ADC and SQCC

**Figure 3:**
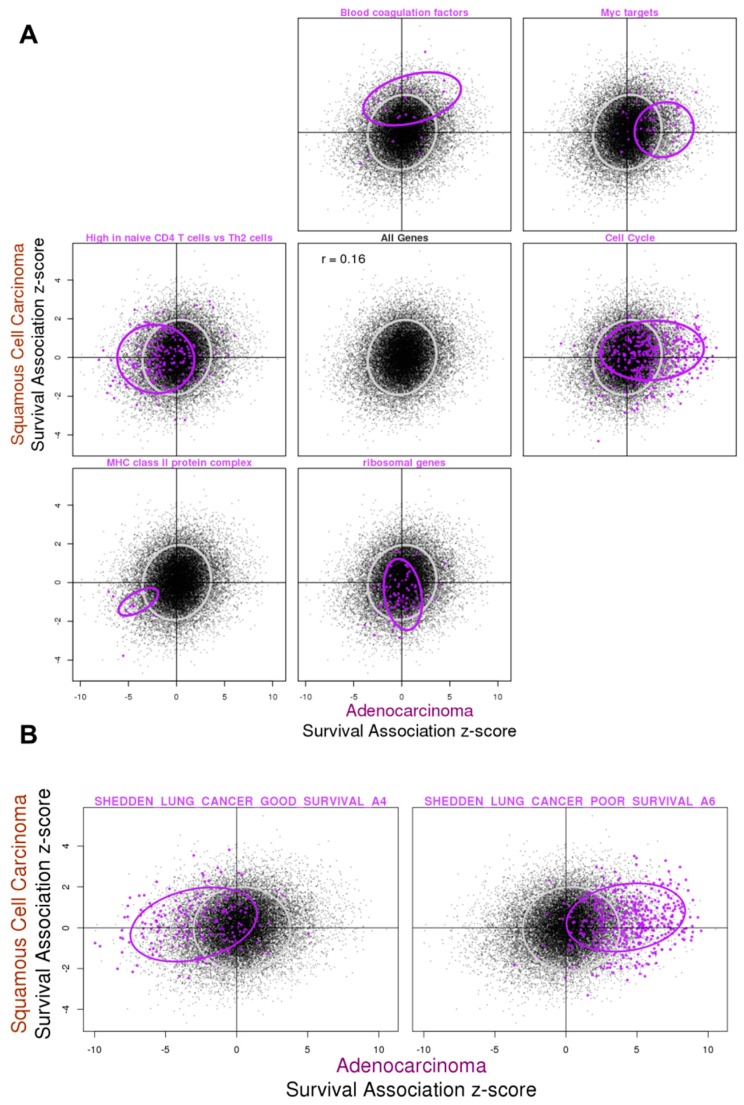
Gene expression survival associations in ADC and SQCC with selected gene sets highlighted. Summary *z*-scores from the meta-analysis of gene expression survival association based on the Cox-PH model were plotted for all genes, with the results from ADC on the *x*-axis and results from SQCC on the *y*-axis. The Pearson correlation coefficient for all genes was 0.16. (**A**) Genes from the selected gene sets coming from the MSigDB gene set collections were highlighted. A selection of gene sets that showed the different patterns for ADC and SQCC association were also highlighted, including: “Blood coagulation factors” from MODULE_131 in the c4 computational gene set collection, “Myc targets” from HALLMARK_MYC_TARGETS_V2 in the hallmark gene set collection, “High in Naïve CD4 T cells vs. Th2 cells” from GSE22886_NAIVE_CD4_TCELL_VS_12H_ACT_TH2_UP in the c7 immunologic signatures gene sets collection, “Cell Cycle” from REACTOME_CELL_CYCLE in c2.cp curated the canonical pathway gene set collection, “MHC class II protein complex components” from GO_MHC_CLASS_II_PROTEIN_COMPLEX in c5.cc GO cellular component gene set collection, and “ribosomal genes” from KEGG_RIBOSOME in the c2.cp curated canonical pathway gene set collection. (**B**) Two prognosis-associated gene sets summarized from a previous lung adenocarcinoma study, SHEDDEN_LUNG_CANCER_GOOD_SURVIVAL_A4 and SHEDDEN_LUNG_CANCER_POOR_SURVIVAL_A6 in the c2.cgp curated chemical and genetic perturbation gene set collection, were also highlighted. Ellipsoidal boundary wraps were around the 75% highest density/minimum volume for all genes (gray) or selected genes (purple).

In the gene-expression survival association analysis, we applied a Cox proportional-hazards model (Cox-PH) to estimate the association between gene expression and overall survival and generated summary statistics from the meta-analysis (Tables S5 and S6). Like the results from the tumor vs. normal gene expression difference analysis, we also observed a positive correlation between the results from ADC and SQCC, but the strength of the correlation appeared much weaker (Figure 3A, center panel). Part of this could potentially be attributed to the poor reproducibility among the individual studies for survival association analysis, especially in SQCC (Figure S3D).

For these results, we also performed pathway analysis with fgsea (Tables S7 and S8). For ADC, gene signatures from a previous study [19] that identified prognostic genes in ADC emerged among the top hits (Figure S6). Highlighting these genes from the said signatures, we observed good consistency with our results, as the worse prognosis set predominantly had positive *z*-scores, whereas the good prognosis set mostly had negative *z*-scores (Figure 3B). Interestingly, this trend was not observed for results from SQCC (Figure 3B and Figure S6).

We also highlighted several gene sets that showed different behavioral patterns in ADC and SQCC (Figure 3A and Figure S6). We saw some histology-specific changes such as the higher expression of genes encoding blood coagulation factors being associated with worse survival, specifically in SQCC. Hypercoagulation in lung cancer has been associated with a higher risk of invasion and metastasis [20], hence potentially explaining this observation as they are also tied to worse prognosis. On the other hand, expression of ribosomal genes seems to be associated with better overall survival specifically in SQCC, without a clear explanation. For ADC-specific changes, we found that cell cycle gene expression was associated with worse survival, consistent with the idea that fast proliferation of the tumor cells is linked to worse prognosis. On the other hand, genes previously found to have higher expression in naïve CD4 T cells when compared to Th2 cells were found associated with better survival in ADC, suggesting that the ratio of infiltrating naïve CD4 T cells over certain differentiated effector T cells might be of prognostic values. Indeed, when we checked several other gene sets from the same study summarizing genes differentially expressed in naïve CD4 T cells and Th1 or Th2 cells, we saw a similar trend for genes highly expressed in naïve CD4 T cells, but the opposite trend for genes with lower expression in naïve CD4 T cells (Figures S7 and S8).

Besides the histological type-specific survival associations, we also found examples of genes that exhibited a similar survival association in ADC and SQCC. For example, the elevated expression of a set of Myc target genes was found associated with worse survival, consistent with the oncogenic role of Myc, while the higher expression of genes encoding components of the class II major histocompatibility complex (MHC II) were observed to be associated with better survival (Figure 3 and Figure S6). The MHC II complex serves to present antigens and initiate immune response, and hence is important for tumor immunosurveillance. MHC II expression has been reported to serve as a marker for favorable response to anti-PD-1 therapy for melanoma and classic Hodgkin lymphoma [21,22]. In lung cancer, MHC II is expressed in both tumor-infiltrating lymphocytes (TILs) and tumor cells, and the expression in tumor cells could be induced by interferon-gamma [23,24]. It has been reported that the expression of MHC II in TILs is associated with the prognosis in lung cancer [25,26]. Although in the present analysis the data were collected from studies conducted prior to the application of immunotherapy to lung cancer, our results revealed the important role of the immunological microenvironment of the tumor in determining the patient outcome.

### 3.4. Relationship between Gene Expression Difference in Tumor vs. Normal and Association with Overall Survival

**Figure 4:**
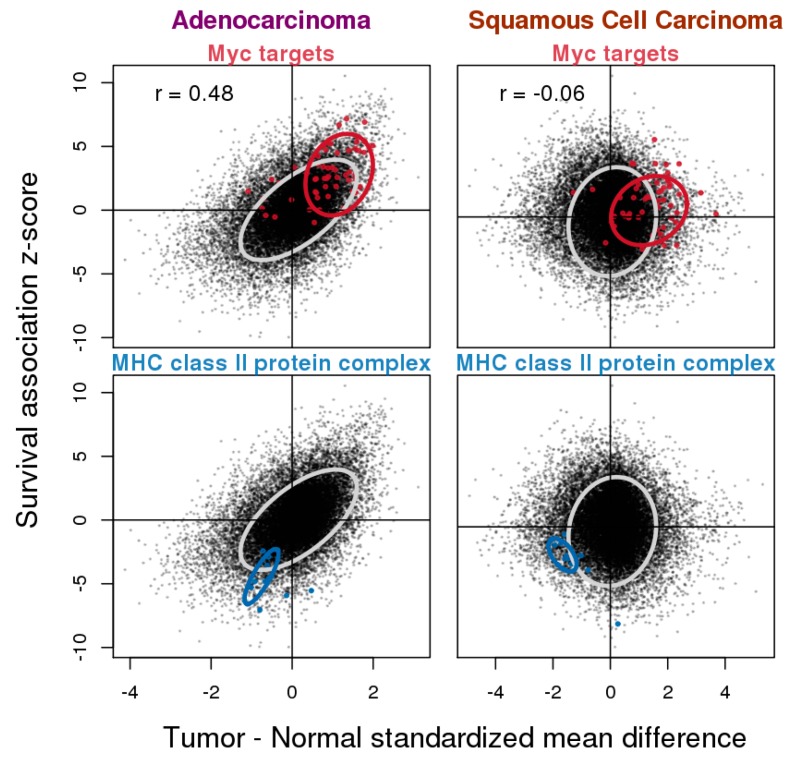
Relationship between tumor–normal gene expression difference and survival association in ADC and SQCC. For all genes, summary standardized tumor–normal gene expression differences from the meta-analysis were used as the *x*-axis values and summary *z*-scores from the survival association meta-analysis based on the Cox-PH model were used as the *y*-axis values. Results from ADC and SQCC were plotted separately. Moderately positive correlation with a Pearson correlation *r* = 0.48 was observed for ADC, while that of SQCC was close to 0. Selected gene sets were highlighted including the Myc targets from HALLMARK_MYC_TARGETS_V2 in the hallmark gene set collection and MHC class II protein complex components in the c5.cc GO cellular component gene set collection. Ellipsoidal boundary wraps were around the 75% highest density/minimum volume for all genes (gray) or selected genes (non-gray colors).

Next, we examined the relationship between the results from the two types of analyses conducted in ADC and SQCC (Figure 4). The tumor vs. normal gene expression difference is commonly used to infer the role that genes play in tumor development. It is generally believed that genes with upregulated expression in tumors tend to adopt oncogenic roles, while genes that are downregulated are tumor suppressive. As expected, we did observe a positive correlation between the tumor vs. normal gene expression difference and the *z*-scores from the survival analysis, but only in ADC (Pearson correlation coefficient *r* = 0.47), while no correlation was observed for SQCC (*r* = −0.01). We highlighted the two example gene sets that were found to be consistently associated with overall survival in both histological types. For Myc target genes that were associated with worse survival, their levels were also higher in tumors when compared to normal tissue samples, and for MHC II complex component genes that were associated with better prognosis, their levels were lower in tumors when compared to normal tissue samples. These examples suggest that despite the moderate to poor global correlation between the tumor vs. normal expression difference and survival association in ADC and SQCC, the correlation is still preserved for certain genes.

### 3.5. A Case Study of FAM83A and FAM83B Expression

In recent years, studies of FAM83A and FAM83B have established them as novel transforming oncogenes that function as intermediaries in EGFR/RAS signaling in different types of cancer [27,28,29,30]. It was recently proposed that *FAM83A* and *FAM83B* could serve as prognostic biomarkers and potential new therapeutic targets in NSCLC based on a study with 362 patients that quantified *FAM83A* and *FAM83B* expression by qPCR [5].

**Figure 5:**
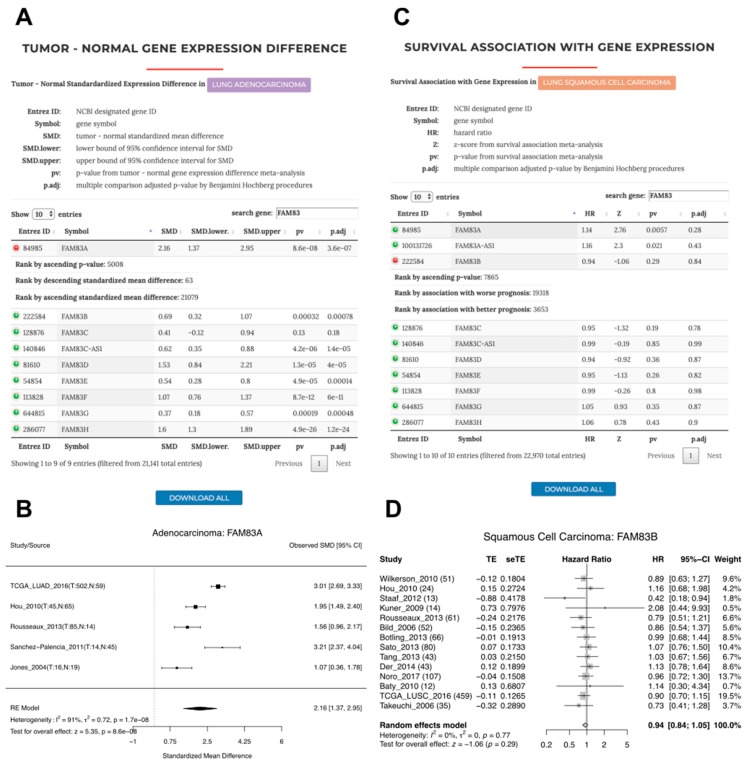
Tumor–normal expression difference of *FAM83A* in ADC and survival association with *FAM83B* expression in SQCC. (**A**) A snapshot of the systematic analysis results for tumor–normal expression difference in ADC on LCE. “FAM83” was entered into the search box so that the table was filtered to retain genes starting with FAM83. (**B**) Forest plot showing meta-analysis of the tumor–normal expression difference in ADC for *FAM83A*. (**C**) A snapshot of the systematic analysis results for the survival association with the FAM83 family gene expression in SQCC on LCE. (**D**) Forest plot showing the meta-analysis of association between *FAM83A* expression and overall survival in SQCC.

As Richtmann reported upregulation of both *FAM83A* and *FAM83B* in ADC as well as SQCC compared to adjacent normal tissue [5], we checked our systematic analysis results table under LCE and verified this relationship (Figure 5A and Figure S9A). The search and filter function under LCE allowed us to put a key in the search box and when we typed “FAM83”, the results for genes starting with FAM83 were retained in the data table, and we obtained a concise view of all FAM83 family members. Consistent with the previous report [27], besides *FAM83A* and *FAM83B*, many other FAM83 genes were also upregulated in tumors. Notably, the effect size for *FAM83A* was the highest among the FAM83 genes and ranked number 63 of the 21,142 genes tested globally for a positive effect size. We also used the meta-analysis tool from LCE to obtain the forest plot as a more fine-grained view to show that *FAM83A* expression was consistently upregulated in ADC tumor across several studies (Figure 5B). As Richtmann et al. [5] also examined the association between *FAM83A* and *FAM83B* and the overall survival in ADC as well as SQCC, we retrieved the overall survival association results in the LCE systematic analysis section (Figure 5C and Figure S9B). We found that both *FAM83A* and *FAM83B* expression were associated with worse survival in ADC (Figure S9B), but in SQCC, only *FAM83A* was associated with worse survival, whereas the result for *FAM83B* was non-significant (Figure 5C,D). This again agrees with the observations from Richtmann et al. [5]. Interestingly, a previous study by Okabe et al. presented a different finding for *FAM83B* survival association in SQCC [31]. Richtmann et al. specifically discussed the discrepancy between findings from the two groups and proposed that the inconsistency could be due to differences in the quantification method (qPCR vs. immunohistochemistry), ethnicity, etc. [5]. As the differences seen by Okabe et al. [31] were in disease-free survival, we additionally performed meta-analysis for studies with recurrence-free survival (RFS) data and showed that although both *FAM83A* and *FAM83B* expression were associated with worse prognosis in RFS, results were non-significant for both genes in SQCC (Figure S10A–D). With these findings (Figure 5D and Figure S10A), we are now more confident that mRNA expression for *FAM83B* would not be a good prognostic marker for SQCC, while it remains to be seen whether the prognostic effects of FAM83B protein levels seen by Okabe et al. [31]. could be validated in additional cohorts. On the other hand, we confirmed the finding shared by both studies that *FAM83B* has higher expression in SQCC compared to ADC with a meta-analysis (Figure S10E).

**Figure 6:**
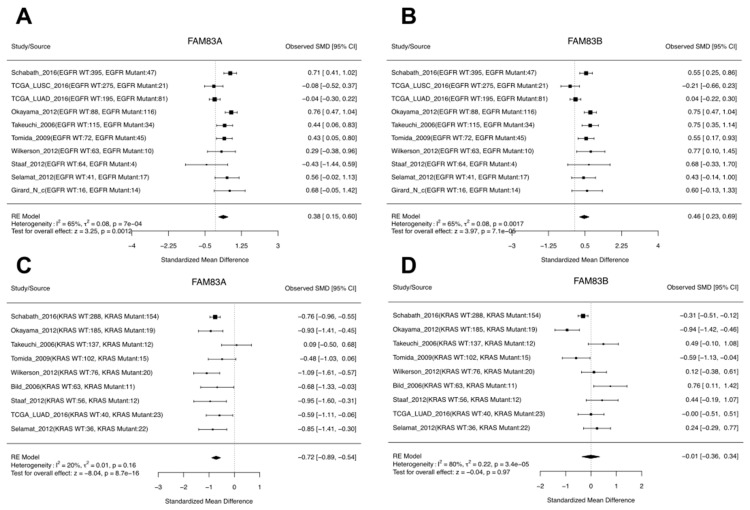
Expression difference between tumors bearing mutations in *EGFR* or *KRAS* compared to WT for *FAM83A* and *FAM83B.* (**A**,**B**) Forest plots showing the *FAM83A* (**A**) and *FAM83B* (**B**) expression difference between tumors with or without *EGFR* mutations. (**C**,**D**) Forest plots showing the *FAM83A* (**C**) and *FAM83B* (**D**) expression difference between tumors with or without *KRAS* mutations.

As *FAM83A* and *FAM8B* have been shown to interact with different components within the *EGFR* pathway and mediate resistance to EGFR inhibitors [29,30], Richtmann et al. [5] also investigated the expression levels of *FAM83A* and *FAM83B* in tumors with *EGFR* mutations compared to those without. They found that *FAM83A* but not *FAM83B* was upregulated in tumors with *EGFR* mutation. From our meta-analyses, we observed that both *FAM83A* and *FAM83B* were higher in tumors with *EGFR* mutations (Figure 6A,B). In addition to *EGFR*, we also checked the expression difference for *FAM83A* and *FAM83B* for tumors with or without *KRAS* mutation by meta-analysis. Interestingly, we found that *FAM83A* but not *FAM83B* was downregulated in tumors bearing *KRAS* mutations (Figure 6C,D) as the *EGFR* mutation, *KRAS* mutation, and *ALK* rearrangement are largely mutually exclusive events in lung cancer [32]. This further indicates that *FAM83A* activation may differ by driver mutation and could serve as an EGFR mutant-specific therapeutic target.

**Figure 7:**
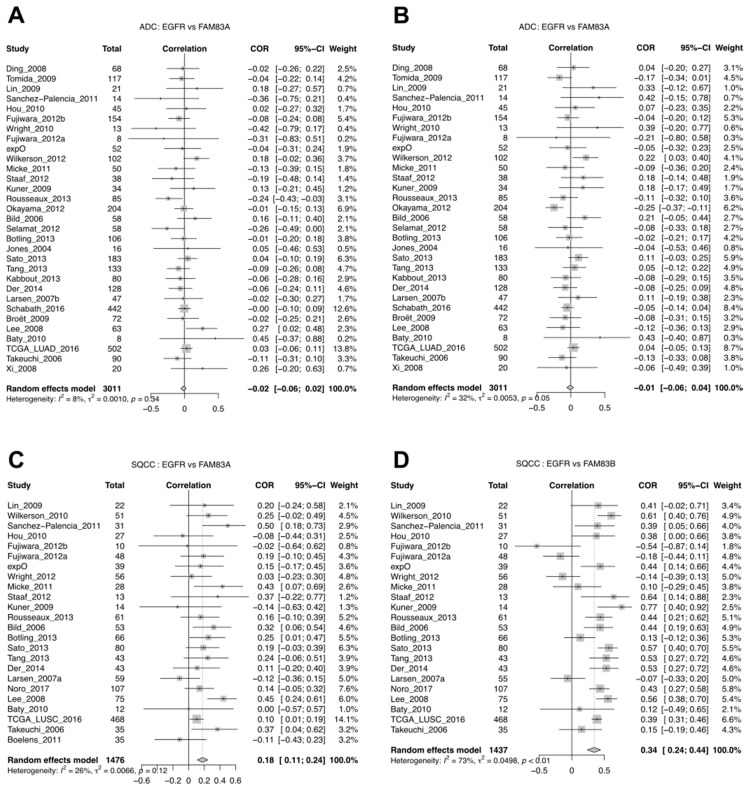
Meta-analysis of correlation in ADC and SQCC for *FAM83A* and *B* vs. *EGFR* expression. (**A**) Forest plot for the correlation between *FAM83A* and *EGFR* in ADC. (**B**) Forest plot for the correlation between *FAM83B* and *EGFR* in ADC. (**C**) Forest plot for the correlation between *FAM83A* and *EGFR* in SQC. (**D**) Forest plot for the correlation between *FAM83B* and *EGFR* in SQC.

Although Richtmann et al. did not find a difference for *FAM83A* or *FAM83B* in tumors with different EGFR mutation status in SQCC, they noticed that the expression of *FAM83A* and *FAM83B* was correlated with *EGFR* expression in SQCC [5]. With a meta-analysis of correlation between *FAM83A* or *FAM83B* and *EGFR* expression, we verified this finding. We saw that in ADC, neither *FAM83A* nor *FAM83B* were correlated with *EGFR* expression in ADC (Figure 7A,B), whereas in SQCC, both *FAM83A* and *FAM83B* were correlated with *EGFR* expression (Figure 7C,D).

## 4. Discussion

When performing survival association with clinical data alone, we observed the expected association between tumor stage, age, and overall survival. Surprisingly, gender-specific prognosis association was only significant for ADC, but not SQCC; this finding nevertheless matches observations from a previously conducted large cohort study. This evidence brought us reassurance in the quality of our clinical data (Figure 1).

In this work, we focused on two types of analyses: gene expression differences in tumors compared to normal tissues, and gene expression association with overall survival. Analyses were done separately for ADC and SQCC, the two main histological subtypes of lung cancer, and the results were compared. We saw a much greater consistency across studies for the tumor vs. normal gene expression difference analysis regardless of histological subtypes, whereas for survival association, the reproducibility in the ADC studies was moderate and the reproducibility in SQCC studies was very poor (Figure S3). Based on the meta-analysis, for ADC and SQCC, the expression differences between the tumor and normal samples were highly similar (Pearson correlation *r* = 0.82, Figure 2), although we did see some SQCC-specific changes. The degree of similarity in the survival association results was much smaller, probably because the analysis was less robust due to the poor reproducibility across individual studies. SQCC predominantly arises in smokers and it has been found that SQCC exhibits fewer cancer-specific deaths compared to ADC [33]. The poor reproducibility in the analysis of overall survival association with gene expression may be explained by the confounding effect of smoking-related comorbidity. Nevertheless, through pathway enrichment analysis, we found that both the histological subtype-unique and -common results (Figure 3). By examining the results from the two types of analyses in conjunction, we observed a positive correlation between the expression changes in tumors when compared to normal samples and worse survival association, and the direction of the changes could be attributed to the oncogenic or tumor suppressive roles of the genes (Figure 4).

In this paper, we made extensive use of pathway analysis to extract biological meanings from the meta-analysis findings. Results of the systematic analyses are provided both as Supplementary Tables for this paper and as downloadable tables from http://lce.biohpc.swmed.edu/lungcancer/, so that readers and LCE users can answer their own questions. On a special note, comparisons between ADC and SQCC have been conducted in previous studies using large datasets such as those from TCGA [34,35]. These studies were carried out from unique angles, but each depended on datasets from a single source, and therefore they could not address the issue of reproducibility. Additionally, our study examined tumor vs. normal gene expression difference in conjunction with survival association, allowing researchers to integrate these two types of evidence to evaluate the oncogenic or tumor suppressive role of their gene of interest. As a case study, we specifically sought to validate findings that originated from a previous publication on the oncogenic role of *FAM83A* and *FAM83B* in NSCLC [5]. Indeed, our meta-analyses results supported most of the findings by Richtmann et al. [5].

In our lung cancer database, we collected and curated 25 clinical variables [1], and numerous ways of systematic analysis could be applied to our data collection. The results we presented in this paper may serve as good examples to inspire more creative ways to perform systematic analysis or validate existing findings with the resource we have built for the lung cancer research community.

## 5. Conclusions

Our previous efforts in constructing a rigorously curated lung cancer database that included both transcriptomic and clinical data from 56 lung cancer studies for over 6700 patients has enabled the creation of a user-friendly web tool that allows gene-based queries and dataset downloads [1]. The establishment of our database also opens up numerous opportunities to conduct genome-wide systematic analysis. Although the data in our collection came from many different sources, our previous efforts in data standardization allowed us to take the meta-analysis approach, which helped us recognize and overcome the reproducibility issues.

In this paper, we performed genome-wide analyses for ADC and SQCC separately on tumor vs. normal expression difference analysis, and survival association analysis. Results are added to the lung cancer explorer web site for user exploration. Pathway analyses revealed results common or unique to different histological subtypes. Capitalizing on our rich database, we studied *FAM83A* and *FAM83B* genes in NSCLC and validated many findings from a previous biomarker study with extensive meta-analyses [5], showcasing the utility of our database for hypothesis testing.

## Supplementary Materials

The following are available online at https://www.mdpi.com/2072-6694/11/6/886/s1, Figure S1: Compare false positive rates by random permutation; Figure S2: Separation of tumor and normal tissue samples from principal component analysis; Figure S3: Reproducibility of tumor–normal expression difference and survival association across different studies from ADC or SQCC; Figure S4: Statistical summary from fgsea for selected gene sets used in pathway enrichment analysis for tumor–normal expression difference results in ADC and SQCC; Figure S5: Evidence of chr3p deletion in lung cancer; Figure S6: fgsea summary statistics for selected gene sets in Figure 3; Figure S7: Distribution of *z*-scores from the survival association analysis for gene sets from GSE22886 that are differentially expressed in naïve CD4 T cells and selected effector T cells; Figure S8: fgsea summary statistics for selected gene sets in Figure S7; Figure S9: Tumor–normal expression difference of FAM83 genes in SQCC and survival association with FAM83 gene expression in ADC; Figure S10: Meta-analysis of recurrence-free survival association for *FAM83A* and *FAM83B* and *FAM83B* expression difference between SQCC and ADC; Table S1: Tumor vs. normal standardized expression difference in ADC, Table S2: Tumor vs. normal standardized expression difference in SQCC, Table S3: Pathway analysis results for tumor vs. normal standardized expression difference in ADC, Table S4: Pathway analysis results for tumor vs. normal standardized expression difference in SQCC, Table S5: Survival association with gene expression in ADC, Table S6: Survival association with gene expression in SQCC, Table S7: Pathway analysis results for survival associations in ADC; Table S8: Pathway analysis results for survival associations in SQCC.
