# Supplementary material for: Systematic Analysis of Gene Expression in Lung Adenocarcinoma and Squamous Cell Carcinoma with a Case Study of FAM83A and FAM83B
Source: Cancers (Basel). 2019 Jun 25;11(6):886. doi: 10.3390/cancers11060886 (PMC6627508; doi:10.3390/cancers11060886)
Supplement: Supplementary file 1 [file cancers-11-00886-s001.zip › cancers-531753-Supplementary Figures.pdf]

Supplementary Materials

Systematic Analysis of Gene Expression in Lung Adenocarcinoma and Squamous Cell Carcinoma with a Case Study of *FAM83A* and *FAM83B*

Ling Cai, Danni Luo, Bo Yao, Donghan M. Yang, ShinYi Lin, Luc Girard, Ralph J. DeBerardinis, John D. Minna, Yang Xie and Guanghua Xiao

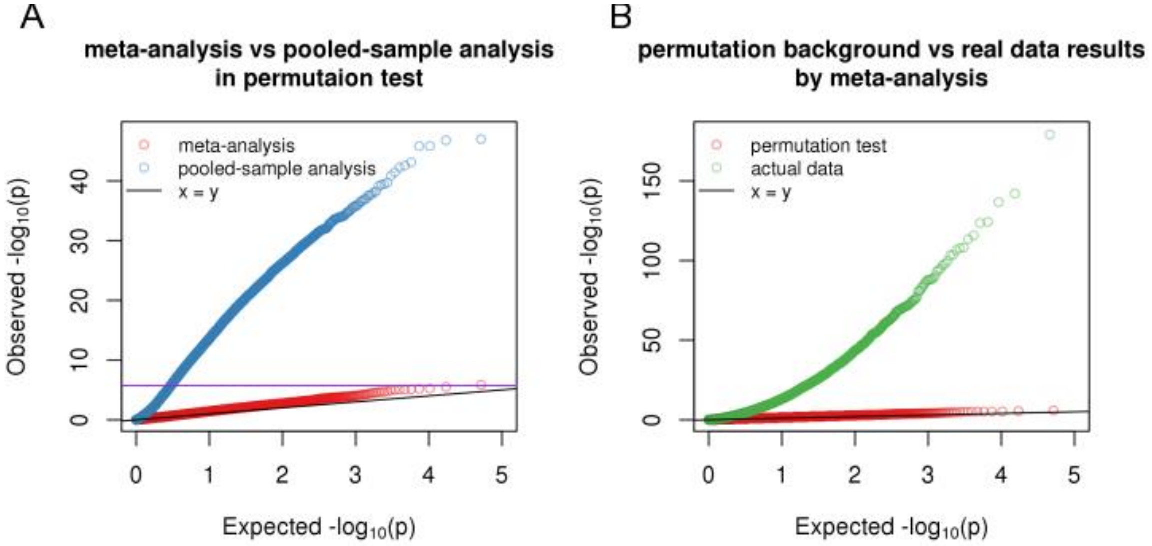

Figure S1. Compare false positive rates by random permutation. (**A**) Compare pooled analysis and meta-analysis in data with randomly permutated labels. We randomly permutated sample labels in the analysis of tumor–normal expression difference. Then, we compared the observed *p*-values generated from the pooled-sample analysis (blue dots, *y*-axis values) and meta-analysis (red dots, *y*-axis values) to their expected uniform *p*-value distribution (*x*-axis values) on a quantile-quantile (QQ) plot. The observed *p*-values from meta-analysis in the permutation test align well with the expected null distribution (by overlapping with the x = y line), indicating that meta-analysis estimates the real statistical significance reasonably well, and leads to few false positives. On the other hand, a large inflation in statistical significance was seen for the pooled-sample analysis, suggesting the existence of many false positives. The purple line indicates the significance=0.05 after Bonferroni correction (i.e., any dots below this line will be identified as hits after Bonferroni correction). (**B**) Compare *p*-values from real observed and randomly permutated data in meta-analysis. We compared the *p*-values generated from the real observed data from the meta-analysis (green dots, *y*-axis values) and *p*-values generated from data with randomly permutated labels. Although a large portion of this observed data was statistically significant, given the comparison we had in the permutation test, we are confident that hits identified by meta-analysis from real observed data are reliable.

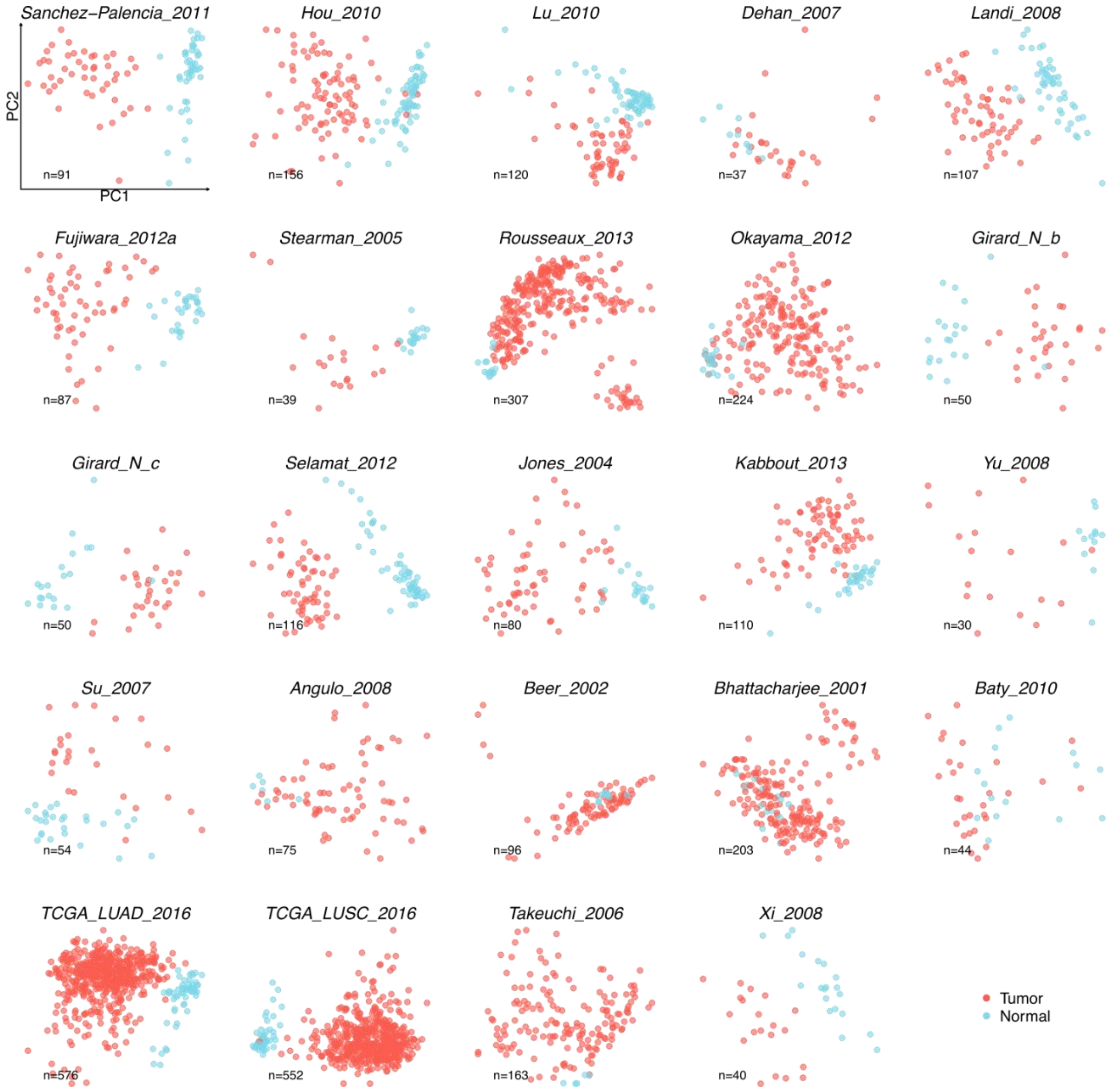

Figure S2. Separation of tumor and normal tissue samples from principal component analysis. We selected datasets from studies with both tumor and non-malignant tissue samples. Principal component analysis was performed and the first two principal components were used for projection of the samples. With the samples colored by the tissue origin types, we found in the majority of the studies, tumor and normal tissue samples were clearly separated. However, the existence of a few exceptions indicates that additional factors other than tissue type exerted stronger influence in driving the transcriptomic differences across all samples.

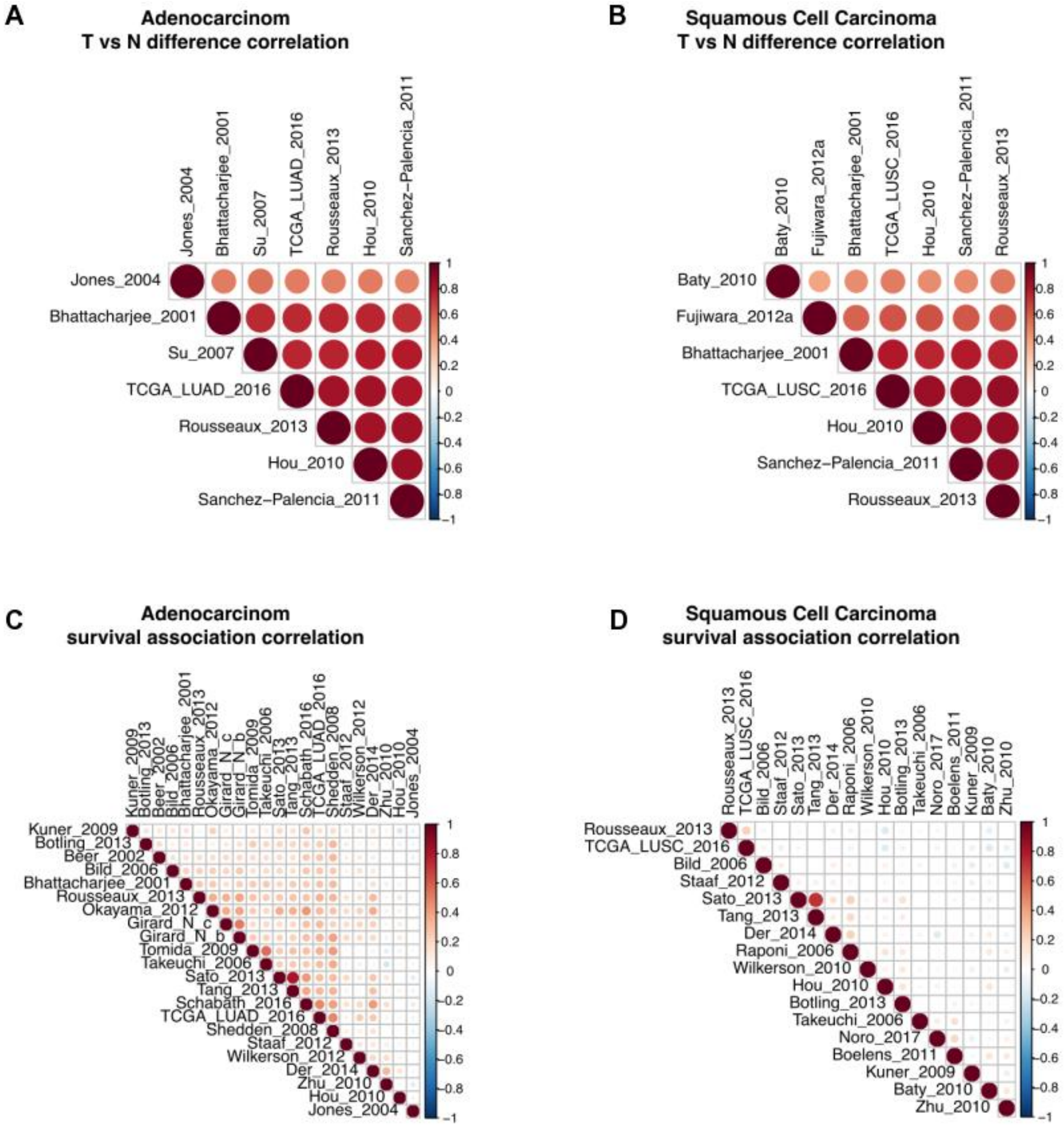

Figure S3. Reproducibility of tumor–normal expression difference and survival association across different studies from ADC or SQCC. (**A**,**B**) Standardized mean expression differences between tumor and normal samples were calculated for all genes, and Spearman rank correlation coefficients for pairwise correlation among individual studies were visualized in the correlation plots for ADC studies (**A**) and SQCC studies (**B**). (**C**,**D**) Likewise, *z*-scores from Cox-PH survival association analysis were used to examine pairwise global correlation among ADC studies (**C**) and SQCC studies (**D**).

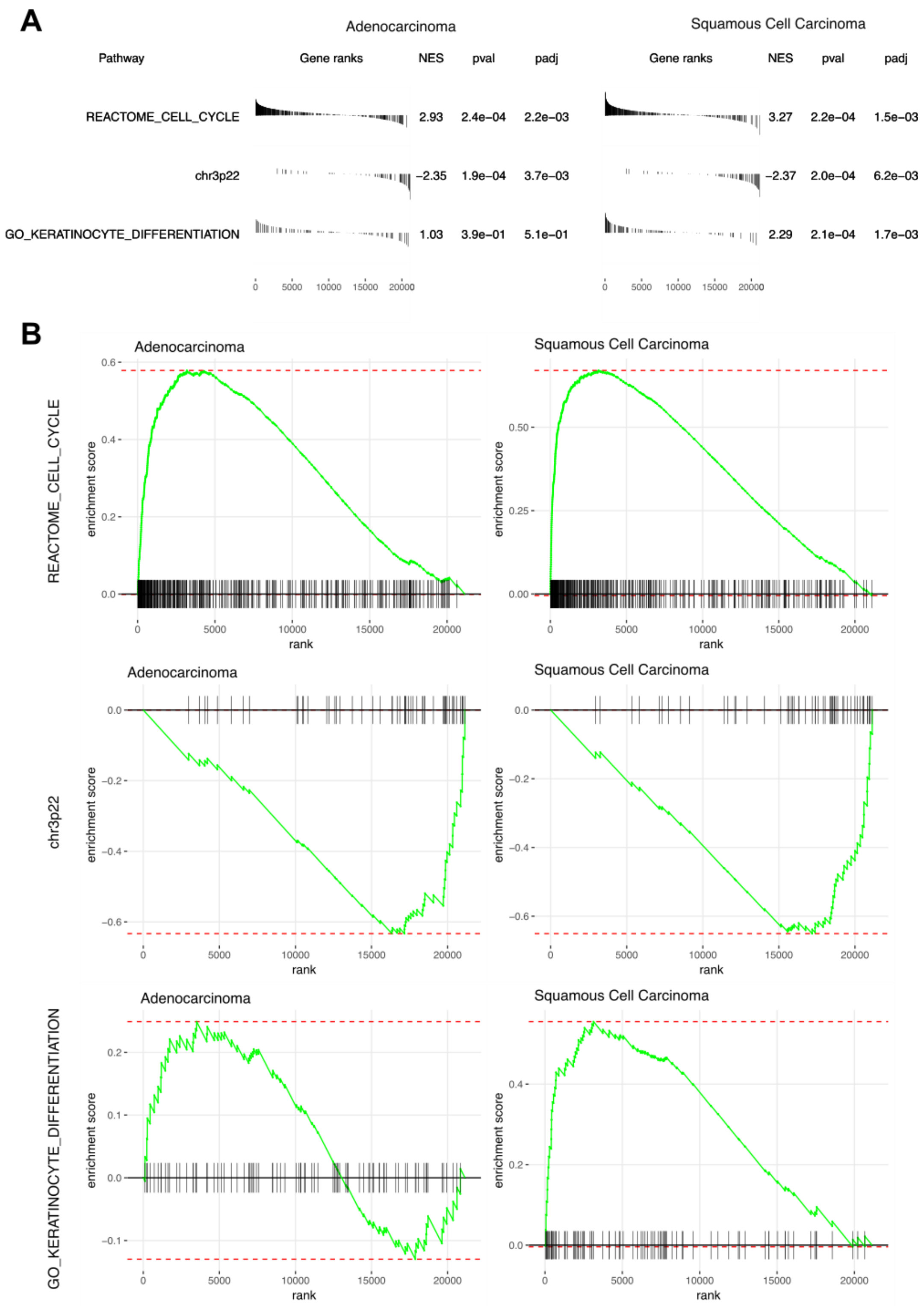

Figure S4. Statistical summary from fgsea for selected gene sets used in pathway enrichment analysis for tumor-normal expression difference results in ADC and SQCC. (**A**,**B**) Normalized enrichment scores (NES), nominal *p*-values and adjusted *p*-values were provided for selected gene sets (**A**). The running enrichment plots were also provided (**B**).

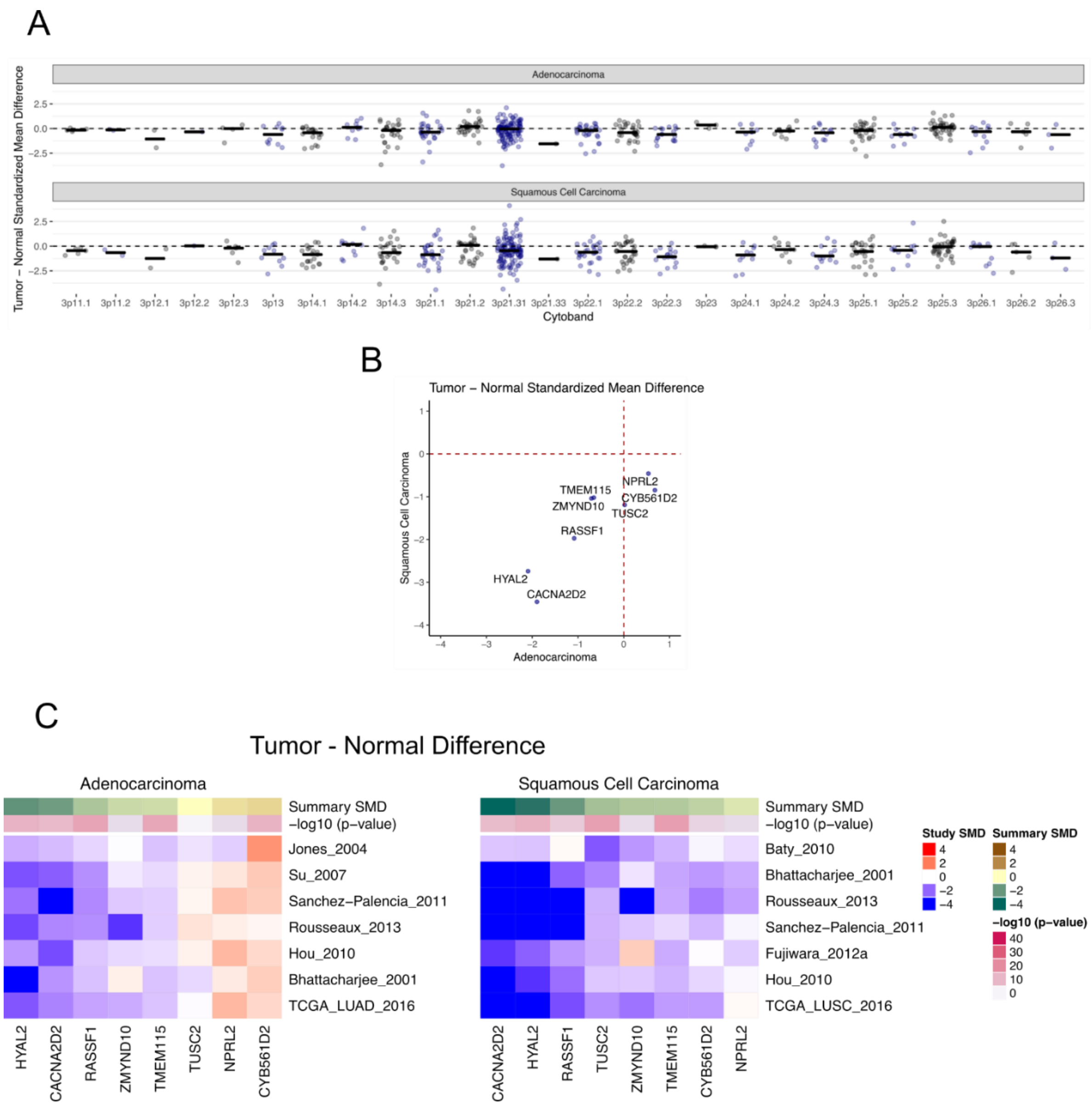

Figure S5. Evidence of chr3p deletion in lung cancer. (**A**) Tumor–Normal expression difference of chr3p genes tends to be negative for ADC and SQCC. Standardized tumor–normal gene expression mean differences from meta-analysis were plotted for genes grouped by their cytoband location on chromosome 3p arm. Black bars represent median SMD for each cytoband. (**B**) Tumor–Normal expression difference from meta-analysis for eight candidate tumor suppressor genes on 3p21.3 in ADC and SQCC. (**C**) Tumor–Normal standardized mean difference for eight candidate tumor suppressor genes on 3p21.3 in different studies of ADC and SQCC.

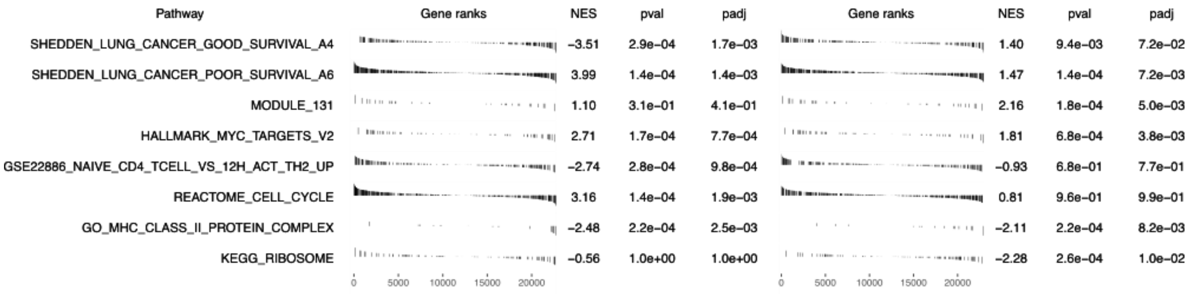

Pathway	NES	pval	padj	NES	pval	padj
SHEDDEN_LUNG_CANCER_GOOD_SURVIVAL_A4	−3.51	2.9e−04	1.7e−03	1.40	9.4e−03	7.2e−02
SHEDDEN_LUNG_CANCER_POOR_SURVIVAL_A6	3.99	1.4e−04	1.4e−03	1.47	1.4e−04	7.2e−03
MODULE_131	1.10	3.1e−01	4.1e−01	2.16	1.8e−04	5.0e−03
HALLMARK_MYC_TARGETS_V2	2.71	1.7e−04	7.7e−04	1.81	6.8e−04	3.8e−03
GSE22886_NAIVE_CD4_TCELL_VS_12H_ACT_TH2_UP	−2.74	2.8e−04	9.8e−04	−0.93	6.8e−01	7.7e−01
REACTOME_CELL_CYCLE	3.16	1.4e−04	1.9e−03	0.81	9.6e−01	9.9e−01
GO_MHC_CLASS_II_PROTEIN_COMPLEX	−2.48	2.2e−04	2.5e−03	−2.11	2.2e−04	8.2e−03
KEGG_RIBOSOME	−0.56	1.0e+00	1.0e+00	−2.28	2.6e−04	1.0e−02

Figure S6. fgsea summary statistics for selected gene sets in Figure 3. Normalized enrichment scores (NES), nominal *p*-values and adjusted *p*-values were provided for selected gene sets used in Figure 3.

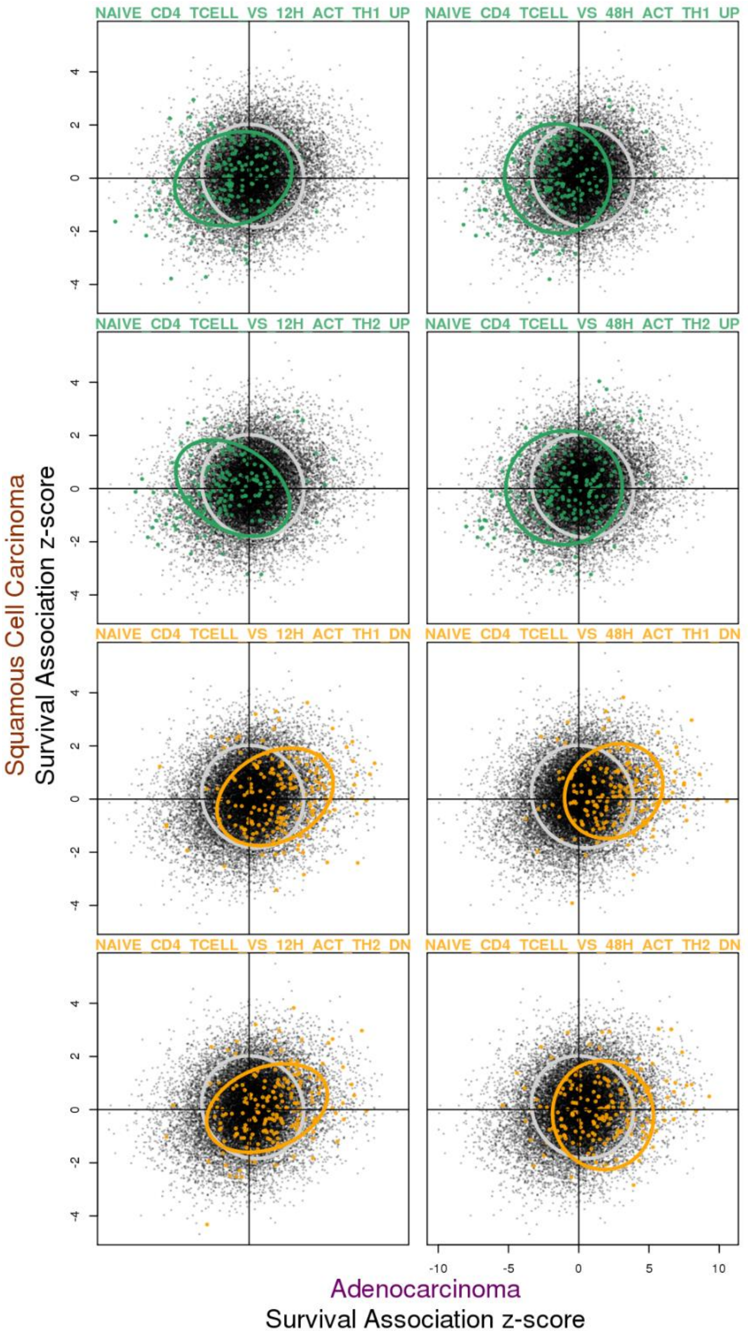

Figure S7. Distribution of *z*-scores from survival association analysis for gene sets from GSE22886 that are differentially expressed in naïve CD4 T cells and selected effector T cells. Gene sets containing genes that changed upon stimulation of naïve CD4 T cells into differentiated effector TH1 or TH2 cells were selected and highlighted on the scatterplot showing *z*-scores from survival association analysis for ADC and SQCC. Genes that were originally high in naïve CD4 T cells tended to associate with more negative *z*-scores in ADC, whereas the opposite is true for genes with lower expression in naïve CD4 T cells. No obvious trend could be observed for SQCC.

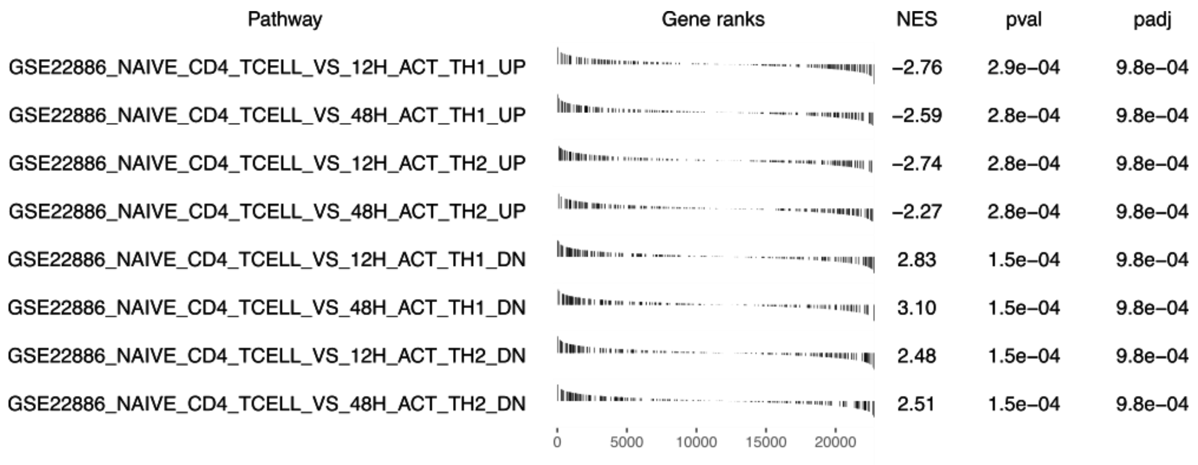

Figure S8. fgsea summary statistics for selected gene sets in Figure S7. Normalized enrichment scores (NES), nominal *p*-values and adjusted *p*-values were provided for selected gene sets used in Figure S7.

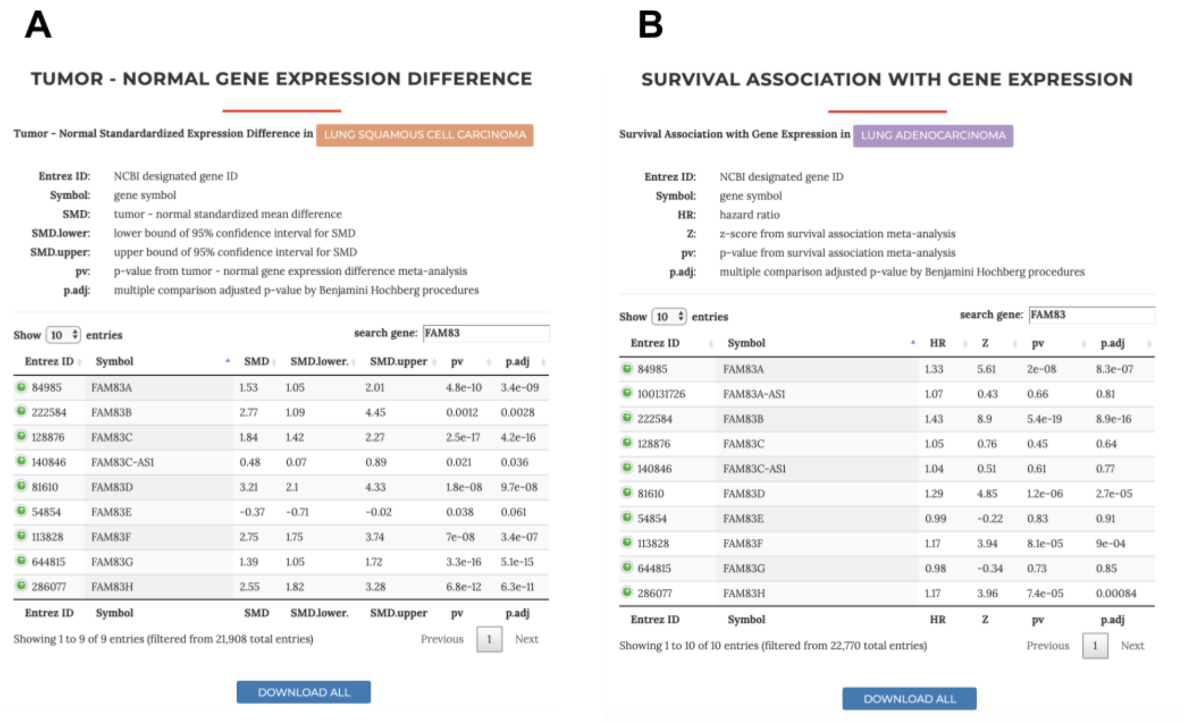

A

TUMOR - NORMAL GENE EXPRESSION DIFFERENCE

Tumor - Normal Standardardized Expression Difference in LUNG SQUAMOUS CELL CARCINOMA

Entrez ID: NCBI designated gene ID
Symbol: gene symbol
SMD: tumor - normal standardized mean difference
SMD.lower: lower bound of 95% confidence interval for SMD
SMD.upper: upper bound of 95% confidence interval for SMD
pv: p-value from tumor - normal gene expression difference meta-analysis
p.adj: multiple comparison adjusted p-value by Benjamini Hochberg procedures

Show 10 entries   search gene: FAM83

Entrez ID	Symbol	SMD	SMD.lower.	SMD.upper	pv	p.adj
84985	FAM83A	1.53	1.05	2.01	4.8e-10	3.4e-09
222584	FAM83B	2.77	1.09	4.45	0.0012	0.0028
128876	FAM83C	1.84	1.42	2.27	2.5e-17	4.2e-16
140846	FAM83C-AS1	0.48	0.07	0.89	0.021	0.036
81610	FAM83D	3.21	2.1	4.33	1.8e-08	9.7e-08
54854	FAM83E	-0.37	-0.71	-0.02	0.038	0.061
113828	FAM83F	2.75	1.75	3.74	7e-08	3.4e-07
644815	FAM83G	1.39	1.05	1.72	3.3e-16	5.1e-15
286077	FAM83H	2.55	1.82	3.28	6.8e-12	6.3e-11
Entrez ID	Symbol	SMD	SMD.lower.	SMD.upper	pv	p.adj

Showing 1 to 9 of 9 entries (filtered from 21,908 total entries)   Previous 1 Next

DOWNLOAD ALL

B

SURVIVAL ASSOCIATION WITH GENE EXPRESSION

Survival Association with Gene Expression in LUNG ADENOCARCINOMA

Entrez ID: NCBI designated gene ID
Symbol: gene symbol
HR: hazard ratio
Z: z-score from survival association meta-analysis
pv: p-value from survival association meta-analysis
p.adj: multiple comparison adjusted p-value by Benjamini Hochberg procedures

Show 10 entries   search gene: FAM83

Entrez ID	Symbol	HR	Z	pv	p.adj
84985	FAM83A	1.33	5.61	2e-08	8.3e-07
100131726	FAM83A-AS1	1.07	0.43	0.66	0.81
222584	FAM83B	1.43	8.9	5.4e-19	8.9e-16
128876	FAM83C	1.05	0.76	0.45	0.64
140846	FAM83C-AS1	1.04	0.51	0.61	0.77
81610	FAM83D	1.29	4.85	1.2e-06	2.7e-05
54854	FAM83E	0.99	-0.22	0.83	0.91
113828	FAM83F	1.17	3.94	8.1e-05	9e-04
644815	FAM83G	0.98	-0.34	0.73	0.85
286077	FAM83H	1.17	3.96	7.4e-05	0.00084
Entrez ID	Symbol	HR	Z	pv	p.adj

Showing 1 to 10 of 10 entries (filtered from 22,770 total entries)   Previous 1 Next

DOWNLOAD ALL

Figure S9. Tumor–normal expression difference of FAM83 genes in SQCC and survival association with FAM83 gene expression in ADC. (**A**) A snapshot of systematic analysis results for Tumor–Normal expression difference in ADC on LCE. (**B**) A snapshot of systematic analysis results for survival association with gene expression in SQCC on LCE.

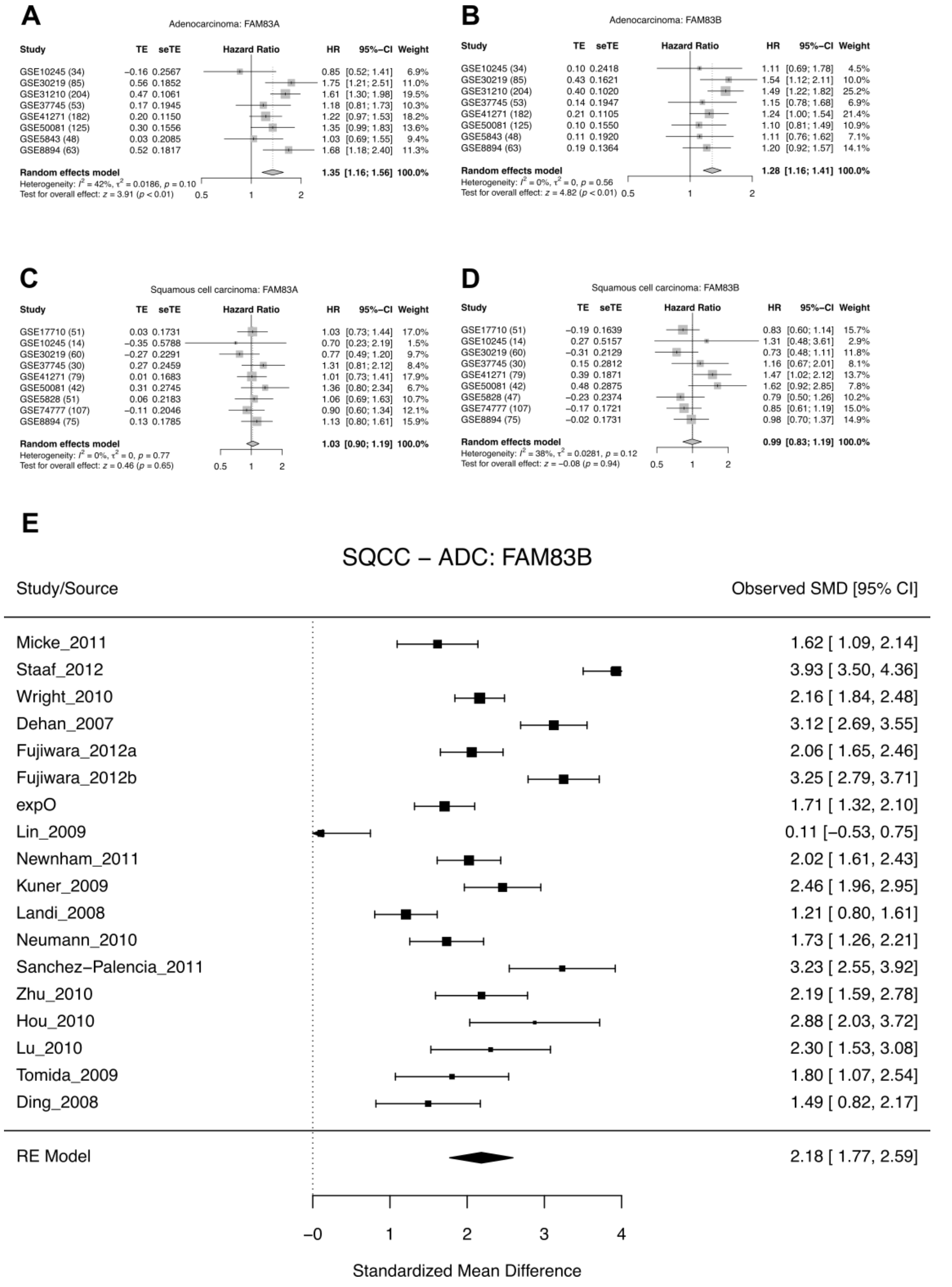

Figure S10. Meta-analysis of recurrence-free survival association for *FAM83A* and *FAM83B* and *FAM83B* expression difference between SQCC and ADC. (**A**,**B**) Forest plots showing meta-analysis of recurrence-free survival with gene expression of *FAM83A* (**A**) or *FAM83B* (**B**) in ADC. (**C**,**D**) Forest plots showing meta-analysis of recurrence-free survival with gene expression of *FAM83A* (**C**) or *FAM83B* (**D**) in SQCC. (**E**) Forest plots showing meta-analysis gene expression difference of *FAM83B* between ADC and SQCC.

© 2019 by the authors. Licensee MDPI, Basel, Switzerland. This article is an open access article distributed under the terms and conditions of the Creative Commons Attribution (CC BY) license (http://creativecommons.org/licenses/by/4.0/).
